# Antioxidant, antibacterial, cytotoxic, and apoptotic activity of stem bark extracts of *Cephalotaxus griffithii *Hook. f

**DOI:** 10.1186/1472-6882-12-30

**Published:** 2012-04-03

**Authors:** Dinesh Singh Moirangthem, Narayan Chandra Talukdar, Naresh Kasoju, Utpal Bora

**Affiliations:** 1 Institute of Bioresources and Sustainable Development, Department of Biotechnology, Government of India, Takyelpat Institutional Area, Imphal 795001, Manipur, India; 2 Indian Institute of Technology Guwahati, Department of Biotechnology, Guwahati 781039, Assam, India

**Keywords:** *Cephalotaxus griffithii*, Polyphenol, Antioxidant, Antibacterial, Cytotoxicity, Apoptosis

## Abstract

**Background:**

*Cephalotaxus *spp. are known to possess various therapeutic potentials. *Cephalotaxus griffithii*, however, has not been evaluated for its biological potential. The reason may be the remoteness and inaccessibility of the habitat where it is distributed. The main aim of this study was to: (1) evaluate multiple biological potentials of stem bark of *C. griffithii*, and (2) identify solvent extract of stem bark of *C. griffithii *to find the one with the highest specific biological activity.

**Methods:**

Dried powder of stem bark of *C. griffithii *was exhaustively extracted serially by soaking in petroleum ether, acetone and methanol to fractionate the chemical constituents into individual fractions or extracts. The extracts were tested for total phenolic and flavonoid content, antioxidant (DPPH radical scavenging, superoxide radical scavenging, and reducing power models), antibacterial (disc diffusion assay on six bacterial strains), cytotoxic (MTT assay on HeLa cells), and apoptotic activity (fluorescence microscopy, DNA fragmentation assay, and flow cytometry on HeLa cells).

**Results:**

Among the three extracts of stem bark of *C. griffithii*, the acetone extract contained the highest amount of total phenolics and flavonoids and showed maximum antioxidant, antibacterial, cytotoxic (IC_50 _of 35.5 ± 0.6 μg/ml; P < 0.05), and apoptotic (46.3 ± 3.6% sub-G0/G1 population; P < 0.05) activity, followed by the methanol and petroleum ether extracts. However, there was no significant difference observed in IC_50 _values (DPPH scavenging assay) of the acetone and methanol extracts and the positive control (ascorbic acid). In contrast, superoxide radical scavenging assay-based antioxidant activity (IC_50_) of the acetone and methanol extracts was significantly lower than the positive control (P < 0.05). Correlation analysis suggested that phenolic and flavonoid content present in stem bark of *C. griffithii *extracts was responsible for the high antioxidant, cytotoxic, and apoptotic activity (P < 0.05).

**Conclusions:**

Stem bark of *C. griffithii *has multiple biological effects. These results call for further chemical characterization of acetone extract of stem bark of *C. griffithii *for specific bioactivity.

## Background

*Cephalotaxus griffithii *Hook. f., a gymnosperm belonging to the family Cephalotaxaceae, is commonly known as Griffith's plum yew. A shrub or small tree, it is found up to an altitude of 2000 m and is distributed in northeastern India, western Sichuan province in China, and Myanmar [1]. Traditional healers of Manipur, a northeastern state of India, use tablets made from *C. griffithii *bark to treat cancer. *Cephalotaxus *spp. have previously been reported to exhibit various biological activities including anticancer [2], osteoblast differentiation [3] and antioxidant activity [4]. It has also been reported that the flavonoids present in *Cephalotaxus *spp. were mainly responsible for such biological activities [2-4]. However, research on *C. griffithii *has been very limited. This may be because of the remoteness and limited accessibility of the habitat of this species. So far, only two phytochemical analyses from *C. griffithii *have been attempted. Kamil et al. [5] isolated and characterized six flavonoids, and Phutdhawong et al. [6] carried out chemical analysis of volatile oil from needles of *C. griffithii*. To our knowledge, to date no data are available on the biological effects of phytochemicals extracted from *C. griffithii*. The main aim of this study was therefore to: (1) evaluate the biological potential of stem bark of *Cephalotaxus griffithii *(SBCG), and (2) identify solvent extract of stem bark of *C. griffithii *to find the one with the highest specific biological activity.

## Results

### Extraction yield, total phenolic and flavonoid content

Table 1 shows the extraction yield, total phenolic content (TPC), and total flavonoid content (TFC) of SBCG extracts. The TPC and TFC in the three extracts were in a range of 72.5 ± 5.1 mg to 609.6 ± 10.1 mg gallic acid equivalents (GAE)/g dried extract and 7.6 ± 0.6 mg to 19 ± 0.6 mg quercetin equivalents (QE)/g dried extract, respectively, with the highest content found in the acetone (ACE) extract followed by the methanol (MeOH) and petroleum ether (PE) extracts (P < 0.05).

**Table 1:** Extraction yield, TPC, and TFC of SBCG extracts

Extract	Yield (% w/w)	Total phenolic content (mg GAE/g extract)	Total flavonoid content (mg QE/g extract)
Petroleum ether	0.9	72.5 ± 5.1a	7.6 ± 0.6a
Acetone	11.2	609.6 ± 10.1b	19 ± 0.6b
Methanol	6.3	420.7 ± 14.5c	11.2 ± 0.3c

Mean ± SD (n = 3)

Means marked with different letters, within each column, are significantly different (*P *< 0.05)

### Antioxidant activity

**Figure 1:**
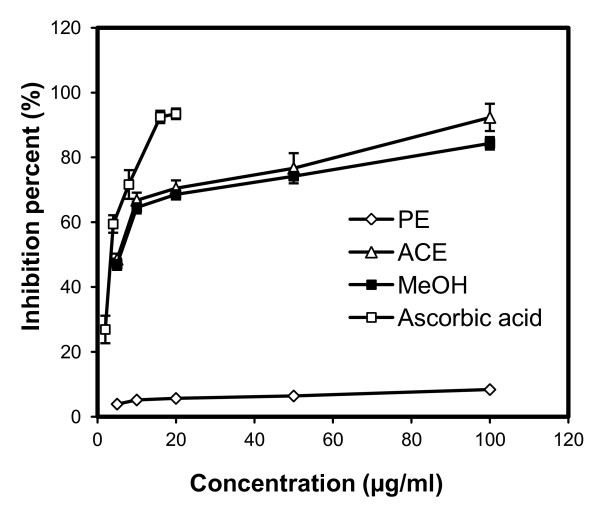
**Antioxidant activity of the PE, ACE, and MeOH extracts of SBCG and ascorbic acid (positive control) assessed by the DPPH radical scavenging method**. Each value represents the mean ± SD of three determinations.

**Figure 2:**
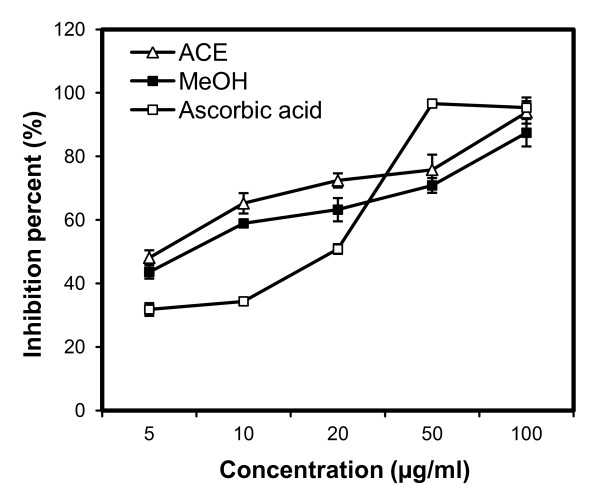
**Antioxidant activity of the ACE and MeOH extracts of SBCG and ascorbic acid (positive control) assessed by the superoxide oxide radical scavenging method**. Each value represents the mean ± SD of three determinations.

**Figure 3:**
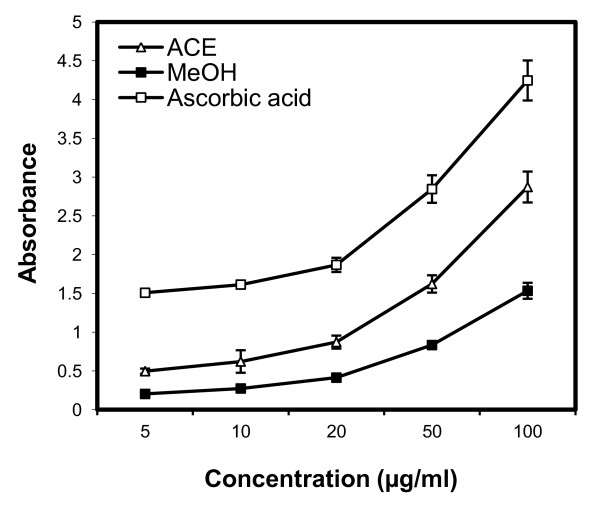
**Antioxidant activity of the ACE and MeOH extracts of SBCG and ascorbic acid (positive control) assessed by the reducing power method**. Each value represents the mean ± SD of three determinations.

Antioxidant potency was tested for DPPH radical scavenging activity, superoxide radical scavenging (SORS) activity, and reducing power, with results shown in Figures 1, 2 and 3. Irrespective of the extracts, DPPH radical scavenging activity was found to be concentration dependent (Figure 1) with highest activity in the ACE extract followed by the MeOH and PE extracts. Table 2 shows the IC_50 _values for the positive control (ascorbic acid) and the three extracts, with a lower IC_50 _indicating greater scavenging power. No significant difference was found between the DPPH IC_50 _values of the ACE and MeOH extracts of SBCG and the positive control. Figure 2 shows SORS activity of SBCG extract and ascorbic acid. The SORS activity was dose dependent. SORS data for the PE extract were not recorded due to precipitation. The ACE extract possessed higher SORS activity than the MeOH extract. The IC_50 _values of these two SBCG extracts were significantly lower than that of the control antioxidant (ascorbic acid) (P < 0.05) (Table 2). The reducing power activity was also dose dependent (Figure 3) with the highest activity found in ascorbic acid. Reducing power of the PE extract was also not recorded because of precipitation, and the ACE extract exhibited higher reducing power than the MeOH extract.

**Table 2:** IC_50 _values of different antioxidant assays of SBCG extracts

Extract	DPPH radical scavenging (μg/ml)	Superoxide radical scavenging (μg/ml)
Petroleum ether	1139.6 ± 33.3a	ND
Acetone	5.3 ± 0.4b	5.5 ± 0.7a
Methanol	5.8 ± 0.5b	7.09 ± 0.5a
Ascorbic acid	3.4 ± 0.15b	19.18 ± 0.49b

Data expressed as mean ± SD (n = 3)

Means marked with different letters, within each column, are significantly different (*P *< 0.05). (ND = not determined)

### Antibacterial activity

**Figure 4:**
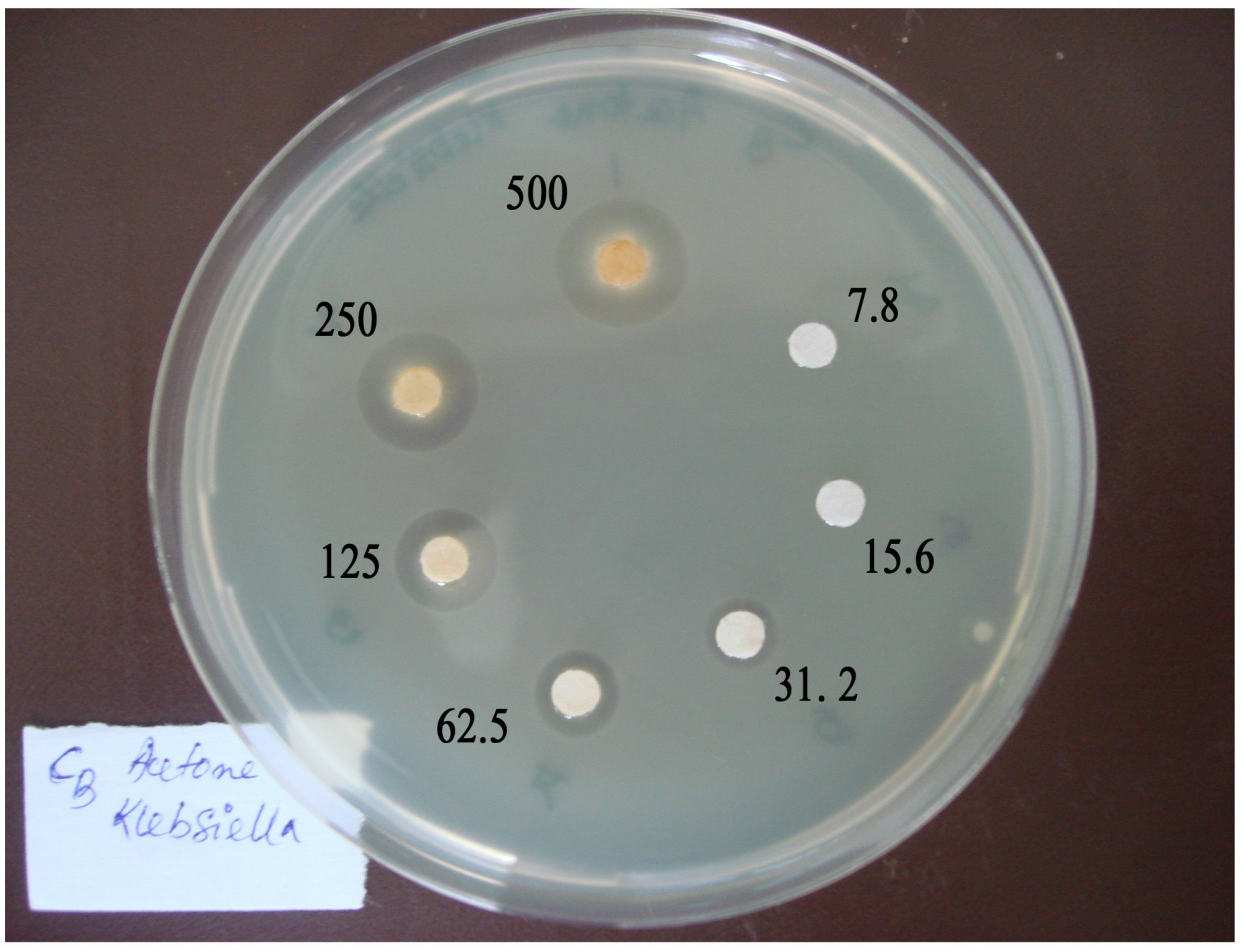
**Appearance of the inhibition zones of *Klebsiella pneumoniae *as a result of application of graded doses (7.8-500 μg/disc) of the ACE extract of SBCG**.

Table 3 shows the inhibition zone diameter of six bacterial pathogens due to the application of two reference antibiotics (neomycin and penicillin) and the three extracts at concentrations of 4 μg/disc and 1000 μg/disc, respectively. The ACE extract produced the largest diameter of inhibition zone (10.8-15.6 mm). Figure 4 shows the inhibition zone of one test organism, *Klebsiella pneumoniae*, towards a graded dose of ACE extract. The negative control showed no inhibition zone (data not shown). The minimum inhibitory dose (MID) of the ACE and MeOH extracts for the three sensitive pathogens were found to be in the range of 31.2-62.5 μg/disc and 125 μg/disc, respectively (Table 4). The PE extract inhibited only *Klebsiella pneumoniae *and the MID was 500 μg/disc.

**Table 3:** Antibacterial activity of different SBCG extracts

Bacteria	Inhibition zone diameter (mm)				
	
	PE (1000 μg/disc)	ACE (1000 μg/disc)	MeOH (1000 μg/disc)	Neomycin (4 μg/disc)	Penicillin (4 μg/disc)
*Klebsiella pneumoniae*	7.1 ± 0.28	15.6 ± 0.2	9.16 ± 0.2	14.5 ± 0.0	ND
*Pseudomonas aeruginosa*	0	0	0	14.1 ± 0.2	ND
*Escherichia coli*	0	10.83 ± 0.2	8.83 ± 0.2	14.1 ± 0.2	ND
*Bacillus subtilis*	0	0	0	9 ± 0.0	ND
*Bacillus cereus*	0	0	0	7 ± 0.0	ND
*Staphylococcus aureus*	0	10.8 ± 0.7	8.8 ± 0.2	0	12 ± 0.0

Data expressed as mean ± SD (*n *= 3).

(ND = not determined)

**Table 4:** MIDs of different positive SBCG extracts

Bacteria	Minimum inhibitory dose (μg/disc)				
	
	PE	ACE	MeOH	Neomycin	Penicillin
*Klebsiella pneumoniae*	500	31.2	125	0.25	ND
*Pseudomonas aeruginosa*	ND	ND	ND	0.5	ND
*Escherichia coli*	ND	62.5	125	0.25	ND
*Bacillus subtilis*	ND	ND	ND	0.5	ND
*Bacillus cereus*	ND	ND	ND	1	ND
*Staphylococcus aureus*	ND	31.2	125	ND	0.25

Data expressed as mean ± SD (*n *= 3).

(ND = not determined).

### Effects of different extracts on the proliferation of HeLa cells

**Figure 5:**
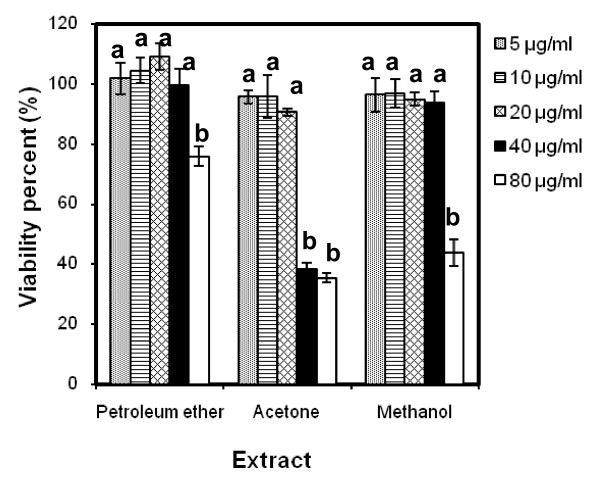
**Cytotoxic effect of the PE, ACE, and MeOH extracts of SBCG against HeLa cells**. Cell survival was determined as the percentage of the control from three independent experiments. Each bar represents the mean ± SD (n = 3). Bars marked without a common letter within each extract are significantly different (P < 0.05).

Figure 5 shows the percentage of viable HeLa cells after treatment with the three SBCG extracts. The relative number of surviving cells decreased in a dose-dependent manner, although there was no significant difference observed between the four lowest concentrations in PE, three lowest concentrations and two highest concentrations in the ACE extract, and four lowest concentrations in MeOH extract. Treatment with the ACE extract produced the greatest concentration-dependent response. For example, ACE extract at 5-80 μg/ml decreased the proliferation of HeLa cells by 95% to 35%. On the other hand, 5-80 μg/ml of either the MeOH or PE extract decreased the viable cells by 96% to 43% and 101% to 75%, respectively. Correspondingly, the IC_50 _value of the ACE extract (35.5 ± 0.6 μg/ml) was found to be the lowest compared with the MeOH extract (75.01 ± 3.4 μg/ml) and PE extract (156.07 ± 5.7 μg/ml) (P < 0.05). The positive control (curcumin) was found to have an IC_50 _value of 6.8 ± 1.4 μg/ml (data not shown).

### Effects of different extracts on inducing apoptosis of HeLa cells

**Figure 6:**
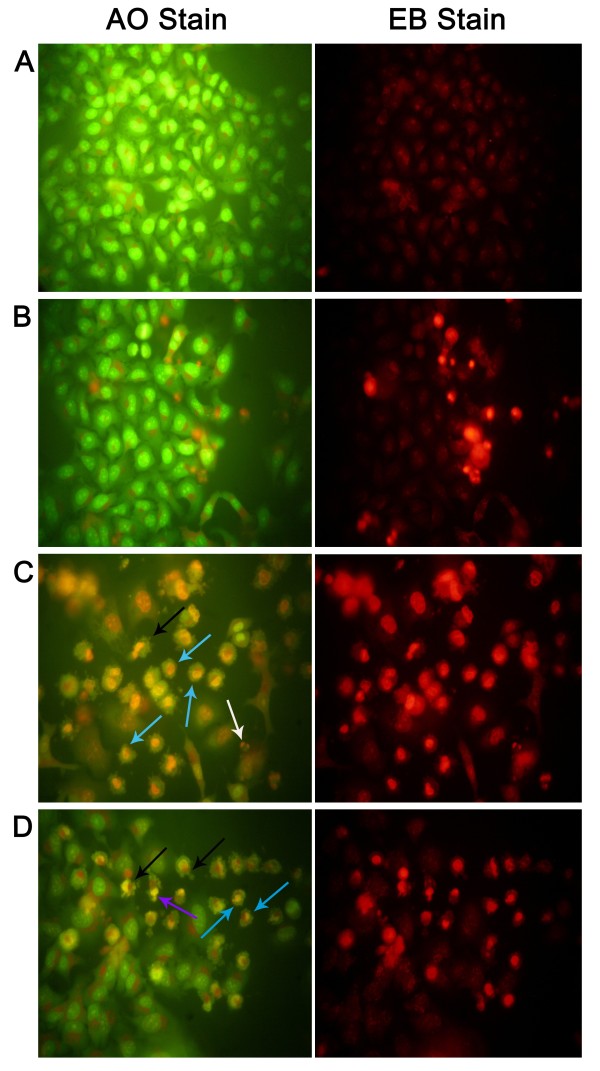
**HeLa cells stained with AO/EB and viewed under fluorescence microscope (400×) showing apoptosis**. (A) Healthy control cells; (B, C and D) Treatment with the PE, ACE, and MeOH extracts (80 μg/ml) on HeLa cells showing membrane blebbing (black arrow), nuclear condensation (blue arrow), budding to form apoptotic body (purple arrow), nuclear fragmentation (white arrow).

**Figure 7:**
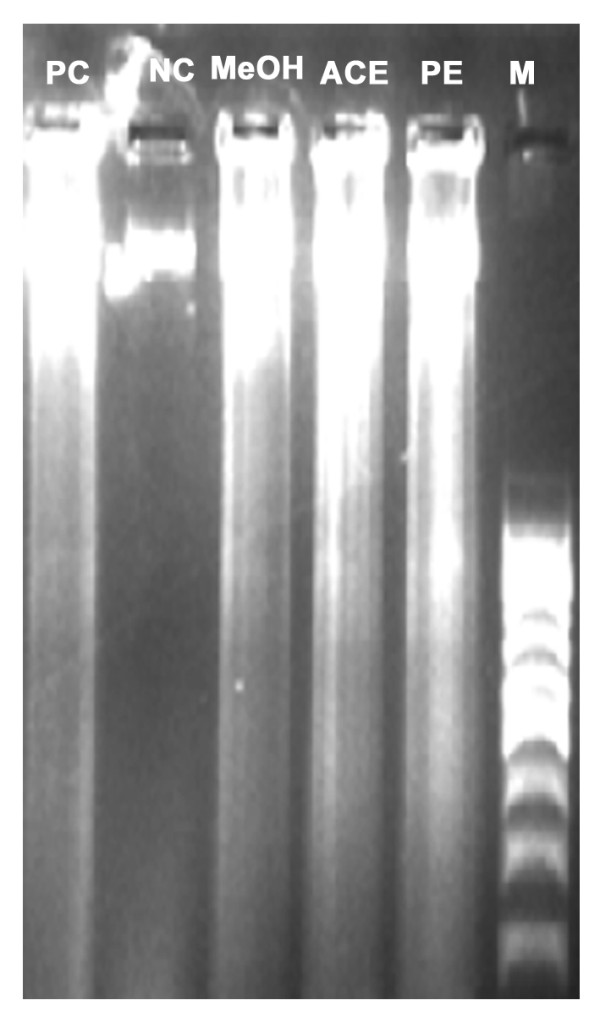
**Gel electrophoresis image obtained after DNA fragmentation assay for apoptosis detection**. The lanes PC, NC, PE, ACE, MeOH, and M represent treatment with the positive control (curcumin), negative control (no treatment), petroleum ether extract, acetone extract, methanol extract (IC_50_), and marker (100 bp DNA ladder), respectively.

Figure 6 shows the appearance of stained HeLa cells under fluorescence microscopy after treatment with the three extracts. The density of HeLa cells, emitting green fluorescence, was lowest due to treatment with the ACE extract, followed by that due to treatment with the MeOH and PE extracts. In other words, there were more dead cells (showing red fluorescence due to AO stain) in the ACE extract treatment group than in the MeOH and PE extract treatment groups. Characteristic features of apoptotic cells, such as cytoplasmic membrane blebbing, nuclear contraction, nuclear fragmentation, and contact inhibition, were visible at 24 h after treatment with the extracts. These features were more prominent in the cells treated with the ACE and MeOH extracts compared with the other treatments (Figure 6C-D). Figure 7 shows results of a DNA fragmentation assay on HeLa cells. DNA fragmentation of extract-treated HeLa cells was comparable with that of cells treated with curcumin, the positive control. Due to a technical reason, a 100-kb ladder marker was used although a 1-kb ladder would have been ideal. Table 5 shows results of the flow cytometry analysis. The sub-G0/G1 population (of HeLa cells) as a biochemical marker of apoptosis was considerably higher in all the extract-treated groups compared with the untreated control. The ACE extract resulted in the highest population of 46.3 ± 3.6% in (P < 0.05) followed by the MeOH extract (35.2 ± 4.4%) (P < 0.05) and then the PE extract (9.1 ± 0.8%). The population of cells in other phases (G1, S, and G2) of the cell cycle were lower, irrespective of the extract (data not shown).

**Table 5:** Effect of SBCG extracts (80 μg/ml) on sub-G0/G1 in HeLa cells

Treatment	Sub G0-G1 population (%)
Untreated	2.9 ± 0.6a
Petroleum ether	9.1 ± 0.8a
Acetone	46.3 ± 3.6b
Methanol	35.2 ± 4.4c

Data expressed as mean ± SD (n = 3)

Means marked with different letters, within each column, are significantly different (*P *< 0.05)

### Correlation analysis

As shown in Table 6 we observed a statistically significant correlation between total phenolic and flavonoid content of the three SBCG extracts and their corresponding DPPH IC_50_, MTT IC_50_, and sub-G0/G1 population (P < 0.05).

**Table 6:** Simple correlations (r value) between TPC and TFC in the SBCG extracts and their DPPH IC_50 _and MTT IC_50 _values and sub-G0/G1 population

Correlations	*r *(*P *< 0.05)
TPC and DPPH IC_50_	0.936*
TFC and DPPH IC_50_	0.742*
TPC and MTT IC_50_	0.995*
TFC and MTT IC_50_	0.915*
TPC and sub-G0/G1 population	0.982*
TFC and sub-G0/G1 population	0.894*

*Statistically significant at *P *< 0.05

## Discussion

This is the first report on the total phenolic and flavonoid content, antioxidant, antibacterial, cytotoxic, and apoptotic activities of stem bark extracts of *C. griffithii*. The total content of phenolic and flavonoid was highest in the ACE extract followed by the MeOH and PE extracts. Earlier studies reported the occurrence of various flavonoids in other species of *Cephalotaxus *[2,7,8]. Polyphenols and flavonoids of several medicinal plants including *Cephalotaxus *spp. are known to exhibit multiple biological effects [2-4,9-11]. Therefore, we investigated the polyphenol and flavonoid enriched SBCG extracts for their various biological activities.

The tested extracts and positive control showed antioxidant activity in a dose-dependent manner. Antioxidant activity varied among the extracts and controls. The highest antioxidant activity was observed in the positive control, and among the three extracts the ACE extract showed the highest antioxidant activity followed by the MeOH and PE extracts. However, there was no statistically significant difference in the DPPH IC_50 _values among the ACE and MeOH extracts and the positive control. In contrast, the IC_50 _value of SORS activity was significantly (Table 2; P < 0.05) lower in both the ACE and MeOH extracts compared with the positive control. In several studies, direct relationships have been observed between total phenolic/flavonoid content and plants' antioxidant activity [12-14]. Many studies also showed potential of purified polyphenols in reducing oxidative stress induced by many factors [15-17]. Bae et al. [4] isolated five flavonoids from *C. koreana*, of which four possessed high levels of antioxidant activity based on DPPH radical scavenging and superoxide radical scavenging assays. In agreement with those reports we observed here a statistically significant correlation between phenolic (r = 0.936, P < 0.05)/flavonoid (r = 0.742, P < 0.05) content and the IC_50 _value of DPPH. This result suggests the presence of potential polyphenols and flavonoids as antioxidants in the SBCG extracts. Antioxidant activity of plant extracts containing polyphenol components is due to their capacity to be donors of hydrogen atoms or electrons and to capture free radicals [18].

The SBCG extracts exhibited differential bacterial inhibitory effects and both the ACE and MeOH extracts inhibited only three pathogens, namely *Klebsiella pneumoniae, Escherichia coli*, and *Staphylococcus aureus*, out of the six organisms tested. The PE extract inhibited only one strain, *Klebsiella pneumoniae*. The ACE extract was the most effective among the extracts as it produced a thicker inhibition zone (10.8-15.6 mm) and smaller MID (31.2-62.5 μg/disc) compared with that produced by the other extracts. Polyphenols are well documented for their antibacterial activities [19,20]. This underscores the importance of the ACE extract of SBCG which showed the highest antibacterial activity and contained the highest amount of polyphenols and flavonoids among the extracts tested. This inhibition of microorganisms by phenolic compounds may be due to iron deprivation or hydrogen bonding with vital proteins such as microbial enzymes or other interactions to inactivate microbial adhesins, cell envelope transport proteins, and/or non-specific interactions with carbohydrates, among other possible effects [19,20]. Earlier, Cho et al. [21] reported an inhibitory effect of Korean plum yew (*C. koreana*) extract on Gram-positive bacteria, Gram-negative bacteria, yeasts, and molds. Similarly, Watanabe and Fukao [22] reported that an extract of unripe Japanese plum yew fruit (*C. harringtonia*) possessed, among 101 edible plants, the highest inhibitory effect against *Bacillus cereus *and *Leuconostoc mesenteroides*. Our results on the antimicrobial effect of SBCG and those of Korean and Japanese plum yew confirm the antibacterial potential of plants in the *Cephalotaxus *genus.

Another important aspect of this study is the observed cytotoxicity of three solvent SBCG extracts on HeLa cells and the apoptotic activity of the extracts. Results of an MTT assay with SBCG indicated that the ACE extract was most effective in inducing cytotoxicity of HeLa cells. This was evident from the lowest IC_50 _(35.5 ± 0.6 μg/ml) of the ACE extract among the three extracts. In a previous study, a methylated biflavone, taiwanhomoflavone-A, isolated from *C. wilsoniana*, was found to possess high antiproliferative activity on KB epidermoid carcinoma of the nasopharynx, COLO-205 colon carcinoma, Hepa-3B hepatoma, and HeLa cervix tumor cells [2], with ED_50 _values of 3.4, 1.0, 2.0, and 2.5 μg/ml, respectively. Similarly, two biflavonoids, namely ginkgetin and 4', 7″-Di-O-methyl-amentoflavone, from *C. koreana *were reported to show cytotoxicity against mouse osteoblasts [3]. There are 1100 publications reporting anticancer activities of polyphenols in the peer-reviewed journals [23] indicating that polyphenols are the main phytochemicals of higher plants possessing antiproliferative properties. We observed a statistically significant correlation between TPC (r = 0.995, P < 0.05)/TFC (r = 0.915, P < 0.05) and MTT IC_50_, which explains the association of the high cytotoxic activity with polyphenol/flavonoid content in the SBCG. It is of interest to design future experiments to identify pure polyphenol/flavonoid molecules in SBCG with anticancer activities. To ascertain whether the cytotoxicity against HeLa cells was mediated through apoptosis, morphological, biochemical, and sub-G0/G1 population studies were carried out on treated HeLa cells. In the morphological study, most of the dead cells showed characteristic features of apoptosis such as cytoplasmic membrane blebbing, nuclear contraction, nuclear fragmentation, and contact inhibition (Figure 6) due to treatment with the extracts. These features were very prominent in the ACE and MeOH extracts. Biochemically, apoptosis is characterized by activation of endogenous nucleases and DNA degradation into fragment multiples of 185 bp [24] which we observed in the DNA fragmentation assay (Figure 7). Furthermore, flow cytometry analysis of treated HeLa cells also showed that the sub-G0/G1 population, a biochemical marker of apoptosis [25] with hypo-diploid DNA, was significantly higher due to treatment with the ACE extract (46.3 ± 3.6%) than with the MeOH extract (35.2 ± 4.4%) compared with control (P < 0.05). This accumulation directly relates to decreases of the cell populations in other phases of the cell cycle, indicating cell death through interference of the cell program. Previous studies showed that polyphenolic compounds induce apoptosis [26,27]. Our study also showed that an increase in the total phenol/flavonoid content of the SBCG extracts significantly increased the rate of apoptosis as evident from the highly significant correlation of TPC/TFC with the sub-G0/G1 population (Table 6). Polyphenolic compounds induce apoptosis in cancer cells by increasing caspase activity [26,28]; upregulating the expression of p53, Bax, Bak, and Bim; and downregulating the expression of Bcl-2 and Bcl-XL [28].

## Conclusions

Based on our results, we conclude that SBCG extracts contain bioactive components responsible for antioxidant, antibacterial, cytotoxic, and apoptotic activities. The polyphenol and flavonoid content of SBCG appears to be responsible for these biological activities. Among the extracts, the ACE extract was found to contain the highest amount of polyphenols and flavonoids and consequently exhibited the highest level of biological activity. We are currently separating and characterizing the bioactive components of the ACE extract of SBCG to test for potential applications in the human health sector.

## Methods

### Chemicals

Folin-Ciocalteu (FC) reagent; gallic acid; 2, 2-diphenyl-1-picryl hydrazyl (DPPH); nitroblue tetrazolium (NBT); 3-(4, 5-dimethyl-2-thiazolyl)-2, 5-diphenyl-tetrazolium bromide (MTT); acridine orange (AO); propidium iodide (PI); and cell culture chemicals were purchased from Sigma-Aldrich Chemicals Pvt. Ltd. (Mumbai, India). Ascorbic acid, curcumin, neomycin sulphate, penicillin, and riboflavin were purchased from HiMedia Laboratories Pvt. Ltd. (Mumbai, India). Proteinase-K, RNase, and ethidium bromide (EB) were purchased from Bangalore Genei (Bangalore, India). Quercetin was purchased from Ozone International, and the rest of the chemicals and solvents used were of analytical grade.

### Plant material

The SBCG was collected from Kangchup Hills, Manipur, India (N24°52'10″ E093°46'12″) at an elevation of 1534.668 m above sea level. The specimen was identified by Dr Biseshori Thongam, Plant Bioresources Division, Institute of Bioresources and Sustainable Development (IBSD), Manipur, India and by Dr S.K. Verma, National Bureau of Plant Genetics Resources, Meghalaya, India. A voucher specimen (IBSD/C/102) has been deposited to the IBSD herbarium.

### Preparation of SBCG extract

The SBCG was air dried at room temperature and powdered. The powdered bark (100 g) was then exhaustively extracted serially by soaking (which prevents the loss of biological activity of some heat-sensitive ingredients) in PE, ACE, and MeOH in order to fractionate the phytochemical constituents into fractions or extracts. Filtration was performed and the filtrates were concentrated in vacuo using a vacuum rotary evaporator (EYELA, Japan) and finally freeze dried (Thermo, Modulyod). The dried extracts were kept at 4°C until further analysis.

### Test sample preparation

Solutions of the test samples for the entire study of the PE extract were prepared in 1, 4-dioxan, except for the antibacterial study in which the sample was prepared in petroleum ether. The ACE and MeOH extracts test sample solutions used in the different experiments were prepared in DMSO. Reagent solutions were made in distilled water, unless otherwise specified.

### Determination of total phenolic content

The TPC was determined using the FC reagent, following the procedure devised by Singleton and Rossi [29], with some modification. Briefly, 20 μl extract (500 μg/ml) was mixed with 1.58 ml distilled water. To this mixture, 100 μl of FC reagent (1:2 dilutions) was added, followed by further addition of 300 μl sodium carbonate (1.8 M). This was incubated for 2 h at room temperature in the dark. The absorbance was then read at 765 nm using a UV-visible spectrophotometer (UV-1700 Pharmaspec, Shimadzu). The TPC was expressed as GAE in milligrams per gram extract after making a standard graph with five different concentrations of gallic acid (50-500 mg/l).

### Determination of total flavonoid content

The TFC of SBCG extracts was determined by the method described by Woisky and Salatino [30]. To 0.5 ml of each extract (1 mg/ml), 0.5 ml of 0.14 M aluminum chloride ethanol solution was added. After 1 h at room temperature, the absorbance was measured at 420 nm. The TFC was expressed as QE in milligrams per gram extract after making a standard graph with five different concentrations of quercetin (12.5-200 μg/ml).

### Scavenging activity of DPPH radical

The free radical scavenging activity of the crude extracts was evaluated as described by Mensor et al. [31]. A solution of DPPH (0.3 mM) in ethanol was prepared and 1 ml added to different concentrations (5-100 μg/ml) of extract samples. After 30 min incubation at room temperature in the dark, absorbance values were measured at 517 nm in a UV-visible spectrophotometer. Ascorbic acid at different concentrations (2-20 μg/ml) was used as positive controls. The inhibition ratio (%) was calculated as follows:

$$\begin{array}{c} \%\;\text{inhibition}=[\left(\text{Absorbance}\;\text{of}\;\text{control}-\text{Absorbance}\;\text{of}\;\text{test}\;\text{sample}\right)/\\ \text{Absorbance}\;\text{of}\;\text{control}]\times 100. \end{array}$$

### Superoxide radical scavenging activity

Measurement of SORS activity was carried out as described by Duan et al. [32], with a slight modification. Various concentrations (5-100 μg/ml) of test samples were mixed with 200 μl EDTA (0.1 M), 100 μl riboflavin (0.5 mM), 200 μl ethanol, and 100 μl NBT (1.2 mM) and the mixture was diluted to 3 ml with phosphate buffer (pH 7.6). The absorbance was then read at 560 nm after illumination for 15 minutes. Ascorbic acid was used as a positive control. The inhibition ratio (%) was calculated as follows:

$$\begin{array}{c} \%\;\text{inhibition}=[\left(\text{Absorbance}\;\text{of}\;\text{control}-\text{Absorbance}\;\text{of}\;\text{test}\;\text{sample}\right)/\\ \text{Absorbance}\;\text{of}\;\text{control}]\times 100. \end{array}$$

### Reducing power

The reducing power was carried out as described by Oyaizu [33]. Various concentrations (5-100 μg/ml) of test samples were mixed with 2.5 ml phosphate buffer (0.2 M; pH = 6.6) and 2.5 ml potassium ferricyanide (0.03 M). After the mixture was incubated at 50°C for 20 min, 2.5 ml of trichloroacetic acid (0.6 M) was added, and the mixture was centrifuged at 1811 g for 10 min. Supernatant (2.5 ml) was mixed with distilled water (2.5 ml) and 0.5 ml of ferric chloride (6.1 mM). The absorbance was then read at 700 nm. Ascorbic acid was used as a positive control.

### Bacterial strains

The following bacterial strains, procured from Microbial Type Culture Collection (MTCC), Institute of Microbial Technology, Chandigarh, India, were used in the screening: *Bacillus subtilis *(MTCC2451), *Bacillus cereus *(MTCC430), and *Pseudomonas aeruginosa *(MTCC2581). *Escherichia coli, Klebsiella pneumoniae*, and *Staphylococcus aureus *were obtained from Dehradun Medical College, Dehradun, India.

### Preparation of discs

Whatman filter paper (no.1) discs (5 mm diameter) were impregnated with 20 μl of crude extracts to get a concentration of 1000 μg/disc and were kept at 37°C for 24 h. The reference antibiotics (neomycin and penicillin) were prepared at appropriate concentrations (4 μg/disc) to serve as the positive controls and solvents PE, ACE, and MeOH as the negative controls.

### Agar disc diffusion method

A modified agar diffusion method was used to determine antibacterial activity [34]. The bacterial cells suspension, 1 × 10^6 ^cfu/ml, was mixed with sterile nutrient agar and poured into petri dishes to give a solid plate.

The discs were deposited on the surface of inoculated agar plates. The bacterial plates were then incubated for 24 h at 37°C. Inhibition zone diameters around each of the discs (diameter of inhibition zone plus diameter of the disc) were measured and recorded at the end of the incubation time. An average zone of inhibition was calculated for three replicates.

### Minimum inhibitory doses

MID was determined by the disc diffusion method. Discs with graded doses (7.8-500 μg/disc) of extracts were prepared, with each consecutive disc containing double the amount of extract prior to when the agar disc diffusion test was performed. MIDs were also determined for positive control. The MID is the lowest quantity of the sample required to inhibit any visible growth.

### Cell culture

The human cervical adenocarcinoma cell line (HeLa) was obtained from the National Centre for Cell Science (Pune, India). These were grown as monolayer cultures in DMEM (Dulbecco's modified Eagle's medium) supplemented with 10% (v/v) heat-inactivated fetal bovine serum (FBS) and 1% antibiotic antimycotic solution (1000 U/ml penicillin, 10 mg/ml streptomycin sulfate, 5 mg/ml gentamycin, and 25 μg/ml amphotericin-B), and maintained at 37°C in 5% CO_2_/95% air with 90% relative humidity.

### MTT reduction assay on HeLa cells

Cytotoxicity analysis was determined using the MTT assay as reported by Mosmann [35]. HeLa cells grown in T-25 culture flasks were harvested by trypsinization, plated at an approximate density of 1 × 10^5 ^cells/well in 96-well culture plates (Corning, Sigma), and incubated for 24 h to achieve confluency. Next the medium from each well was removed and the cells washed twice with Dulbecco's phosphate buffered saline (PBS). The cells were then exposed to increasing concentrations of extract (5-80 μg/ml). Each well contained 100 μl of serum-free DMEM containing different concentrations of extracts. The cells were then incubated at 37°C in 5% CO_2_/95% air with 90% relative humidity for 24 h. After incubation, the contents were replaced with equal amounts of MTT dissolved in serum-free DMEM (1.2 mM) after which the plates were further incubated for 3 h. The contents were then replaced with equal amounts of DMSO to solubilize the formazan grains formed by viable cells. Finally, the absorbance was read at 570 nm using a multi-well plate reader (Tecan, Infinite M200). The viability percentage was calculated by using the formula below:

$$\begin{array}{c} \text{Viability}\;\%=\text{Absorbance}\;\text{of}\;\text{test}\;\text{sample}\times 100/\text{Absorbance}\;\text{of}\\ \text{solvent}\;\text{control}. \end{array}$$

### Fluorescence microscopy

Differential staining of extract-treated (80 μg/ml; 24 h) and untreated HeLa cells was done using DNA-intercalate fluorescent dyes EB and AO [36], and analyzed under a fluorescence microscope (Nikon, TS 100-F; Tokyo, Japan).

### DNA fragmentation assay

For laddering experiments, HeLa cells (2 × 10^6 ^cells/well) were cultured in 6-well culture plates (Corning, Sigma), treated with extracts (MTT test IC_50 _value) and incubated for 48 h in a CO_2 _incubator. Treated cells were then harvested, washed with ice-cold PBS (pH 7.2), and centrifuged at 0.8 g for 6 min at 4°C. The resulting cell pellet was dispersed in 30 μl of lysis buffer (10 mM EDTA; 50 mM Tris HCl, pH-7.8; 1% SDS) by gentle vortexing. About 4 μl of proteinase-K (10 μg/μl) was then added to the above mixture, followed by incubation at 45°C for 1-2 h. Then, 2 μl of RNase (10 μg/μl) was added to the cell lysates, which were further incubated for 1 h at room temperature. After incubation cell lysates were mixed with 4 μl of 6X DNA sample dye and subjected to run at 2% agarose gel electrophoresis. The gel was then stained with ethidium bromide (0.5 μg/ml) and visualized under a gel documentation system (Bio-Rad, USA).

### Flow cytometry

Apoptotic cells were detected using PI staining [37] of HeLa cells followed by flow cytometry to detect the sub-G0/G1 cell population [38]. Briefly, HeLa cells were treated with or without SBCG extract (80 μg/ml) for 24 h. After treatment, floating and adherent cells were harvested and fixed in ice-cold 70% ethanol overnight at -20°C. Fixed cells were then treated with 0.5 ml of DNA extraction buffer (192 ml of 0.2 M Na_2_HPO_4 _with 8 ml of 0.1% Triton X-100 (v/v)) for 5 min at room temperature. DNA was stained with PI (0.02 mM) and incubated for 1 h in the dark. Flow cytometric analysis was then performed using a flow cytometer (BD FACSCaliber). At least 10,000 cells were analyzed for each sample and data were analyzed and plotted by CellQuest software.

### Statistical analysis

The values are presented as mean ± SD (standard deviation) of triplicate measurements. Multiple comparisons between more than two groups were performed by one-way ANOVA supplemented with Tukey's HSD post hoc test. Values at P < 0.05 were considered to indicate statistical significance. Simple correlation analysis was carried out and significance tested by following standard methods [39].

## Competing interests

The authors declare that they have no competing interests.

## Authors' contributions

DSM: performed the entire experiment and prepared the manuscript. NCT: designed the research plan; supervised and coordinated the study; and corrected the manuscript. NK and UB: assisted, along with DSM, in conducting the cell culture-based experiment. All authors have duly checked and approved the manuscript before submission.

## Pre-publication history

The pre-publication history for this paper can be accessed here:

http://www.biomedcentral.com/1472-6882/12/30/prepub
